# Roles of N-glycans in the polymerization-dependent aggregation of mutant Ig-μ chains in the early secretory pathway

**DOI:** 10.1038/srep41815

**Published:** 2017-02-03

**Authors:** Chiara Giannone, Claudio Fagioli, Caterina Valetti, Roberto Sitia, Tiziana Anelli

**Affiliations:** 1 Division of Genetics and Cell Biology, IRCCS San Raffaele Scientific Institute, Milan, Italy; 2 Department of Experimental Medicine, University of Genoa, Genoa, Italy; 3 University Vita-Salute San Raffaele, Via Olgettina 58, 20132 Milan, Italy

## Abstract

The polymeric structure of secretory IgM allows efficient antigen binding and complement fixation. The available structural models place the N-glycans bound to asparagines 402 and 563 of Ig-μ chains within a densely packed core of native IgM. These glycans are found in the high mannose state also in secreted IgM, suggesting that polymerization hinders them to Golgi processing enzymes. Their absence alters polymerization. Here we investigate their role following the fate of aggregation-prone mutant μ chains lacking the Cμ1 domain (μ∆). Our data reveal that μ∆ lacking 563 glycans (μ∆5) form larger intracellular aggregates than μ∆ and are not secreted. Like μ∆, they sequester ERGIC-53, a lectin previously shown to promote polymerization. In contrast, μ∆ lacking 402 glycans (μ∆4) remain detergent soluble and accumulate in the ER, as does a double mutant devoid of both (μ∆4–5). These results suggest that the two C-terminal Ig-μ glycans shape the polymerization-dependent aggregation by engaging lectins and acting as spacers in the alignment of individual IgM subunits in native polymers.

Over one third of the proteome starts folding in the endoplasmic reticulum (ER)^1^,^2^. The ER teams up with the Golgi and Intermediate Compartment to form a functional unit –the early secretory pathway (ESP)- acting coordinately to couple fidelity and efficiency of protein secretion. Key players are resident ESP chaperones and enzymes that favour and time glycoprotein quality control and transport^3^. Despite the existence of sophisticated proteostatic systems, however, mutations, lack of folding assistants or the unbalanced production of different subunits can generate conditions in which proteins that enter ESP (synthesis and translocation) exceed those exiting from it (secretion and/or degradation), causing traffic jams as in ER Storage Disorders (ERSD)^4^.

Secretory IgM are complex molecules, whose assembly occurs stepwise in the secretory pathway. The first step requires the formation of μ_2_L_2_ “monomers” (Fig. 1), covalently linked by inter-chain disulfide bonds. These rapidly assemble in the ER. μ_2_L_2_ that pass the BiP-dependent checkpoints must then form covalent polymers to negotiate secretion^1^,^4^. In the absence of Ig-J chains, hexamers are formed^5^, in which six monomers are bound via homotypic covalent bonds between cysteines 414 and 575 (Fig. 1). The addition and processing of N-glycans is important for IgM biogenesis and quality control. Ig-μ chains contain 5 N-glycans (171, 332, 395, 402 and 563). While the first three are found in a processed state, N402 and N563 are modified by high-mannose sugars in secreted IgM^6^,^7^,^8^, suggesting that they remain hidden to the glycan processing enzymes as polymers travel through the secretory pathway^9^. Exposure of high-mannose moieties upon antigen binding could be important for the clearance of serum immune complexes^7^.

For polymerization to take place, intra-subunit bonds ought to be prevented. At the same time, μ_2_L_2_ subunits should be aligned to form circular polymers of limited size. Previous studies in reconstituted HeLa cells pointed at ERGIC-53, a hexameric lectin that assists ER-Golgi transport of selected glycoproteins^10^, as a platform for IgM polymerization^11^. Moreover, Ig-μ lacking N563 glycans were shown to form higher order polymers devoid of J chains^12^, suggesting that binding to hexameric ERGIC-53 may favour the closure of planar pentamers with a J chain or hexamers. However, since N563 oligosaccharides become inaccessible upon polymerization^9^, they may also act as spacers limiting the number of subunits that can be incorporated into a polymer. Conversely, the absence of N402 glycans inhibits polymerization^12^,^13^.

Owing to the high mutation rate of immunoglobulins and their abundant production by cells of the B lineage, transport-incompetent variants often accumulate in dilated ESP cisternae, called Russell Bodies (RB)^14^, particularly in Mott myelomas and other plasma cell dyscrasias^15^,^16^,^17^. Over the last years, we developed RB models based on the inducible expression of mutant Ig-μ chains lacking the first constant domain (μ∆)^18^,^19^. In all Ig classes, CH1 domains mediate the association with Ig-L chains. In the absence of L, they bind the ER chaperone BiP^20^. Unassembled H chains are secreted in Heavy Chain Diseases, because they lack CH1 and escape BiP-dependent quality control. HCD can cause kidney damage^21^ because CH1 deletion facilitates aggregation^22^.

Since μ∆ variants that cannot polymerize (e.g. μ∆C575A) do not form RB and are secreted^14^, aggregation depends on polymerization. Accordingly, factors that impact polymerization, e.g. Ero1 or ERp44, modulate RB biogenesis^19^. Also elements working *in cis* play a role, including the N563 and N402 glycans, which are located 12 residues upstream the two cysteines involved in polymerization (C414 and C575 respectively). Since formation of disulphide bonds and polymerization are needed for μ∆ aggregation, we set up to investigate the role of the N402 and N563 glycans in the formation of detergent-insoluble μ∆ deposits. Our results show that their absence inhibits or favours aggregation, respectively. Mutants lacking both remain soluble, suggesting that the 402 glycans favour the accessibility of C575 and C414 and ultimately polymerization, possibly binding ERGIC-53. The 563 glycan could limit the number of μ∆_2_ or μ_2_L_2_ subunits that can be incorporated into planar polymers.

## Results and Discussion

### Deletion of the N563 glycan favours aggregation and prevents secretion of mutant Ig-μ lacking the Cμ1 domain (μ∆)

The absence of the Cμ1 domain, the main interactor with the chaperone BiP^20^, increases the tendency of Ig-μ chains to form detergent-insoluble intracellular deposits^14^,^18^, a phenomenon hereafter referred to as aggregation. In the absence of L chains, μ∆ accumulate mainly in ribosome-free cisternae stained by ERGIC-53, called smooth Russell Bodies (sRB). Since disulfide bonding of different μ∆_2_ dimers via C575 is crucial for aggregation, these findings support the notion that ERGIC-53 can promote IgM polymerization^11^,^23^. Rough RB (rRB) form instead upon assembly with L chains^18^. In the absence of a Cμ1 to dock to, the tendency of the Ig-L constant domain (C_L_) to form homodimers might promote interactions amongst μ∆_2_ complexes in the ER, favouring inter-C575 bonding^18^.

**Figure 2:**
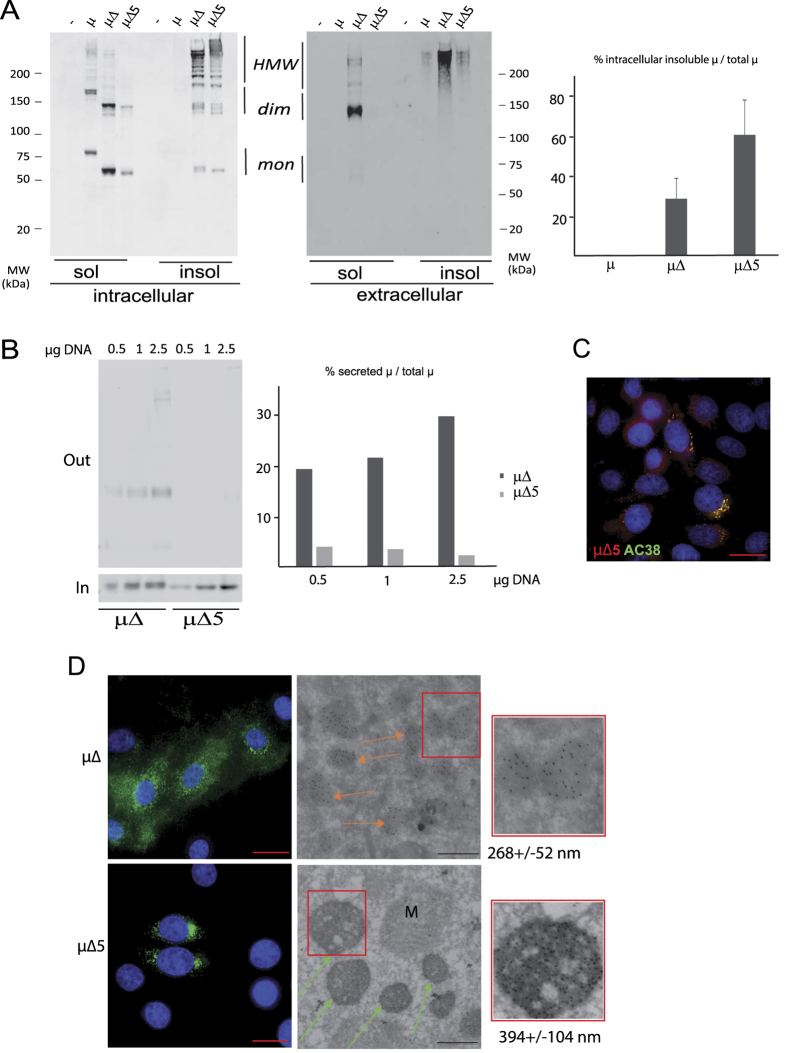
μΔ5 forms bigger aggregates than μΔ. HeLa cells were transiently transfected with vectors encoding for secretory μ, μΔ, μΔ5 or empty vector as a control (−) as indicated. (**A**) The NP-40 detergent-soluble and insoluble material from 10^5^ cells/lane (intracellular, left panel) and the material secreted in 4 hours (sol) or scraped from the plates after removal of the cells with detergent (insol) (extracellular – center panel) was loaded on 4–10% pre-casted polyacrylamide gradient gels under non-reducing conditions and decorated with anti-μ antibodies. More μΔ5 accumulates intracellularly in the NP-40 insoluble fraction, as indicated by the densitomeric quantification shown in the right panel (average of 3 independent experiments +/− standard deviation). (**B**) Total intracellular and extracellular material of HeLa cells transiently transfected with increasing amounts of plasmids encoding for μΔ or μΔ5, as indicated, were loaded on SDS-page and decorated with anti-μ antibodies. The percentage of secreted μ relative to the total intracellular amount was determined by densitometric quantification and is shown in the right panel. (**C**) HeLa cells co-expressing μΔ5 and Ig-λ chains were fixed with PFA and stained with anti-μ (red) and anti-idiotypic antibodies (AC38, green). Co-localization of the AC38 (which recognizes only properly paired μλ complexes) and anti-μ staining confirms that μΔ5 assembles with λ chains (bar: 15 μm). (**D**) HeLa transfectants expressing μ∆ or μ∆5 were fixed with PFA, stained with anti-μ Alexa 488 antibodies and visualized with deconvolution microscopy (left panels, bar: 15 μm). Immunogold analyses (middle and right panels) confirmed that the electron dense material contains condensed μ chains (see arrows) (bar: 500 nm). The enlargements shown in the insets confirm that μ∆5-containing SupeRB are bigger than μ∆-containing sRB. Their diameters (+/− standard deviation) were calculated as the average of 60 such structures analyzed.

When serine 565 is replaced by alanine to destroy the C-terminal NVS glycon, higher order IgM polymers are rapidly formed^9^,^12^,^13^. We thus hypothesized that a double mutant lacking both the Cμ1 domain and the 563 glycan (μ∆5) would show stronger tendency to aggregate. To test this prediction, we expressed μ∆ or μ∆5 in HeLa cells and analyzed their distribution. As expected, much larger amounts of the μ∆5 double mutant accumulated than μ∆ in the detergent-insoluble fraction: furthermore, aggregates contained higher molecular weight covalent complexes (Fig. 2A).

Part of μ∆ chains are secreted by the HeLa cells used in these experiments, either as soluble μ∆_2_ homodimers or as detergent-insoluble high molecular weight complexes that stick to the culture dishes and can be readily stained by immunofluorescence^19^ (see also Fig. 2D below). These complexes contain Endo-H sensitive 402 and 563 glycans, suggesting that they traversed the Golgi in the polymeric state ref. 19 and our unpublished data. Secretion via a Golgi-independent route is less likely, because the 171, 332, 395 glycans are Endo-H resistant.

Only minute amounts of μΔ5 are released extracellularly. The fraction of secreted μ∆ was higher than μ∆5 at all the expression levels tested (Fig. 2B), hence excluding saturation of the retention mechanisms as the sole cause of μ∆ secretion. These results suggest that μΔ5 condense more rapidly and form deposits that cannot be transported further along ESP. When coexpressed with murine Ig-λ, μΔ5 chains react with NP hapten or Ac38 anti-idiotype antibodies (Fig. 2C), confirming proper VH folding and pairing with Vλ. Moreover, in the context of wild-type Ig-μ the A565S mutation does not prevent secretion of hapten binding, Ac38^+^ higher molecular weight polymers, suggesting that the absence of this glycan does not induce gross protein unfolding. Rapid condensation could thus lead to the formation of transport-incompetent large complexes.

Accordingly, immunofluorescence analyses revealed that μ∆5 accumulate in roundish vesicles bigger than sRB (Fig. 2D). This behaviour was not cell specific, as similar detergent solubility and subcellular distribution were obtained in HepG2 or Hek293T transfectants (data not shown). Electron-microscopy analyses revealed that μ∆5 accumulated in deposits with an average diameter larger than what observed for sRB (394 ± 104 vs 268 ± 52 nm), hence the name SupeRB (Fig. 2D). Notably, few if any ribosomes decorated the membrane of SupeRB. Immuno-electron microscopy with gold-coupled anti-μ confirmed the presence of mutant μ chains in both sRB and SupeRB (see arrows).

Thus, preventing the attachment of the most C-terminal N-glycan, which accelerates IgM polymerization, increases the accumulation of μ∆ chains into detergent-insoluble, high molecular weight covalent complexes that deposit in ESP vesicles and are retained intracellularly.

### SupeRB are surrounded by ERGIC-53 positive membranes

**Figure 3:**
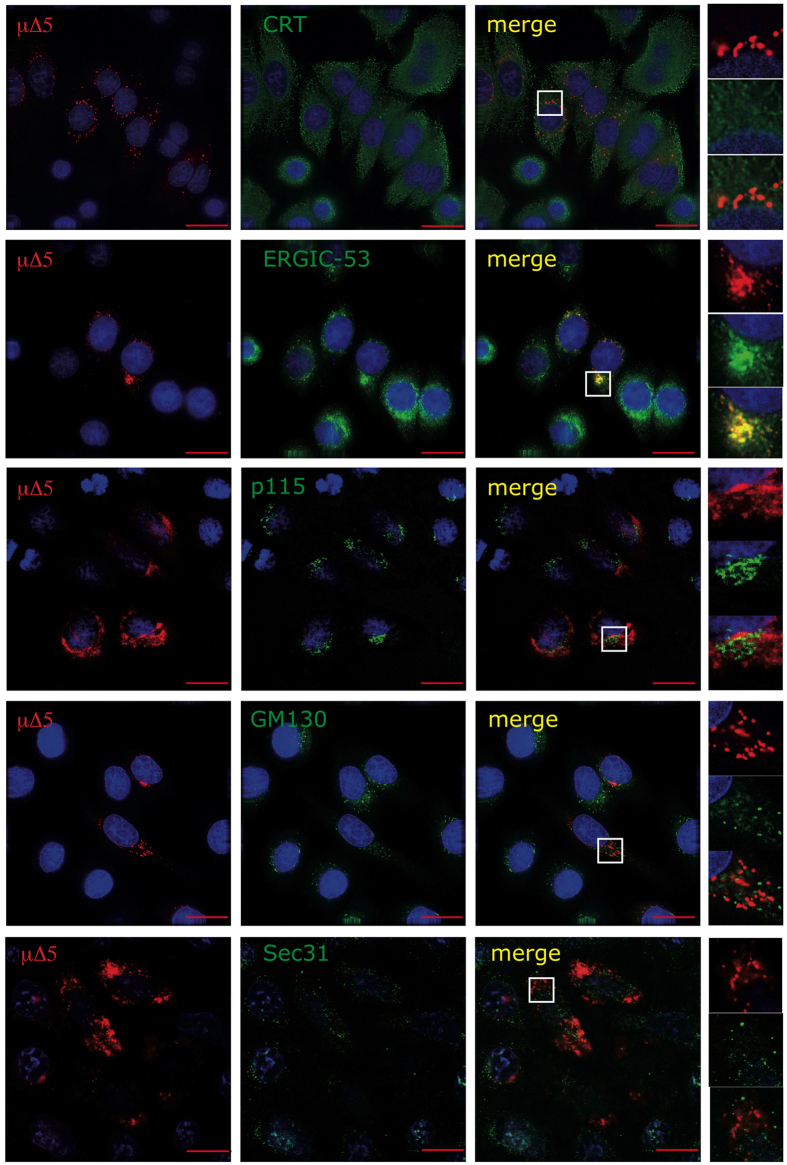
SupeRB contain ERGIC-53. HeLa cells transiently transfected with μΔ5 were fixed with PFA and stained with antibodies against markers of different compartments of the secretory pathway. μΔ5-containing SupeRB clearly co-localize with ERGIC-53 but not with CRT, p115, Sec 31 and GM130. In cells over-expressing μΔ5, ERGIC-53 is itself condensed and recruited to SupeRB (bar: 15 μm).

To investigate the origin and localization of SupeRB, we exploited immunofluorescence co-staining assays. μΔ5-containing SupeRB did not co-localize with the ER marker calreticulin (Fig. 3). The intense co-staining with ERGIC-53 indicated that, similarly to μ∆^18^, μ∆5 bind this lectin, directly or indirectly^13^. Accordingly, ERGIC-53 lost its normal distribution in cells over-expressing μΔ5 and accumulated around SupeRB. Interestingly, SupeRB, as also smooth RBs, do not co-localize with p115 or GM130, markers of late ERGIC^24^ and cisGolgi^25^ respectively (Fig. 3 and Supplementary Figure 1). Sec31, marker of ER exit sites^26^, does not colocalize with μ∆5 (Fig. 3).

**Figure 4:**
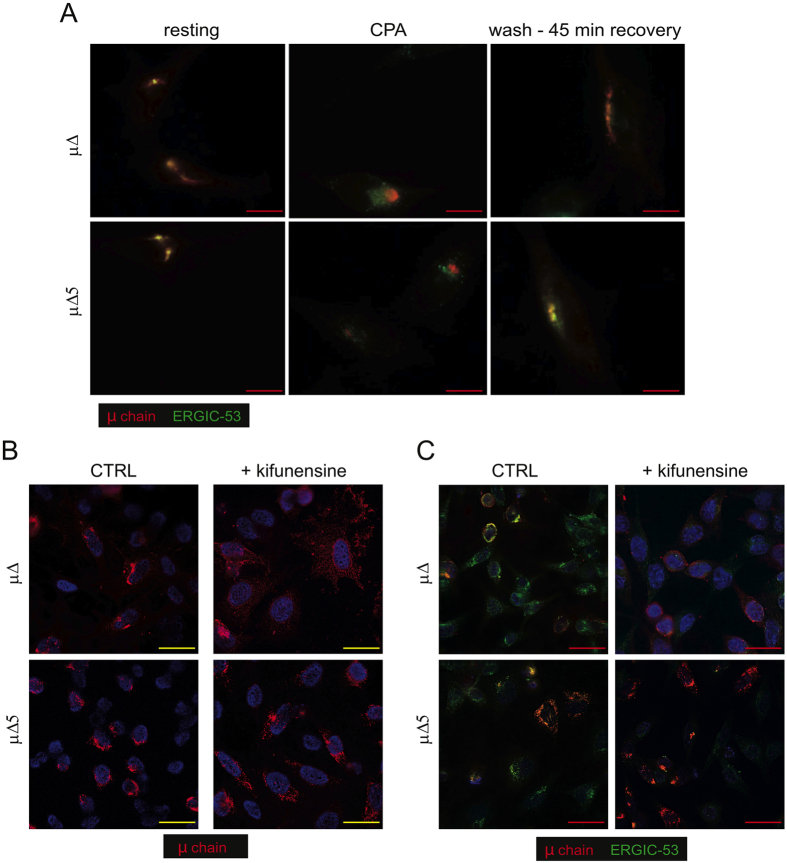
ERGIC-53 establishes lectin-dependent interactions with μΔ and μΔ5. (**A**) Forty hours after transfection, HeLa cells expressing μΔ or μΔ5 (resting cells) were treated with 50 μM CPA. After 2 hours, CPA was washed out and cells were cultured in Ca^2+^ containing medium for 45 minutes before fixation and co-staining with anti-μ (red) and anti-ERGIC-53 (green). Both μΔ and μ∆5 interact with ERGIC-53 in a Ca^2+^-dependent manner (bar: 7 μm). (**B**) Immediately after transfection, HeLa cells expressing μΔ or μΔ5 were treated with or without 21.5 μM kifunensine. After 40 hours, cells were fixed with PFA and stained with anti-μ. In the presence of kifunensine, μΔ no longer aggregates and displays a reticular staining. The phenotype of μΔ5 instead is not modified by kifunensine (bar: 15 μm). **C.** HeLa cells treated as in B were stained with antibodies against μ (red) and ERGIC-53 (green). After treatment with kifunensine, ERGIC-53 looses its co-localization with μΔ but not with μΔ5 (bar: 15 μm).

Since the binding between ERGIC-53 and its glycoprotein ligands is calcium-dependent^27^, treatment with a reversible SERCA inhibitor (cyclopiazonic acid, CPA) weakens lectin-dependent interactions^13^,^18^. Accordingly, ERGIC-53 regained its normal localization upon CPA treatment, even if μ∆ or μ∆5 aggregates remained in place (Fig. 4A). ERGIC-53 associated again with SupeRB upon CPA removal, as previously described for μ∆^18^. The co-localization of ERGIC-53 with μ∆ and μ∆5 could reflect its binding to detergent-insoluble deposits. Whatever their origin, sRB and SupeRB seem to remain in communication with the mainstream secretory pathway: ERGIC-53 can diffuse into them when the affinity of its lectin domains for μ∆ or μ∆5 glycans is higher than the affinity for the cytosolic molecules that normally drive its subcellular localization. Considering the sensitivity of intracellular μ∆ and μ∆5 to Endo-H^19^, sRB and SupeRB seem to originate from the aggregation of soluble cargo molecules between the ER and the Golgi.

### Mannosidase l-dependent trimming of the N563 glycan promotes μ∆ aggregation

We previously showed that kifunensine, an inhibitor of mannosidase I, an enzyme that removes the terminal mannose from the B branch in N-glycans^28^, prevented the aggregation of μ∆ chains in both HeLa and plasma cells^18^. This result suggested a role for a kifunensine-sensitive factor in promoting aggregation, especially considering that the drug would increase the concentration of μ∆ and μ∆5 in ESP^29^,^30^ by preventing their degradation. One such factor could be ERGIC-53^11^,^31^. Unexpectedly, kifunensine had only minor effects on μΔ5 aggregation (Fig. 4B) compared to μ∆, a much larger fraction of μΔ5 becoming detergent-insoluble also under kifunensine treatment. Moreover, in the presence of kifunensine ERGIC-53 lost its co-localization with μΔ, but not with μΔ5 (Fig. 4C), implying the existence of other direct or indirect interactions that are insensitive to kifunensine.

### Removal of the N402 glycan prevents polymerization and aggregation

**Figure 5:**
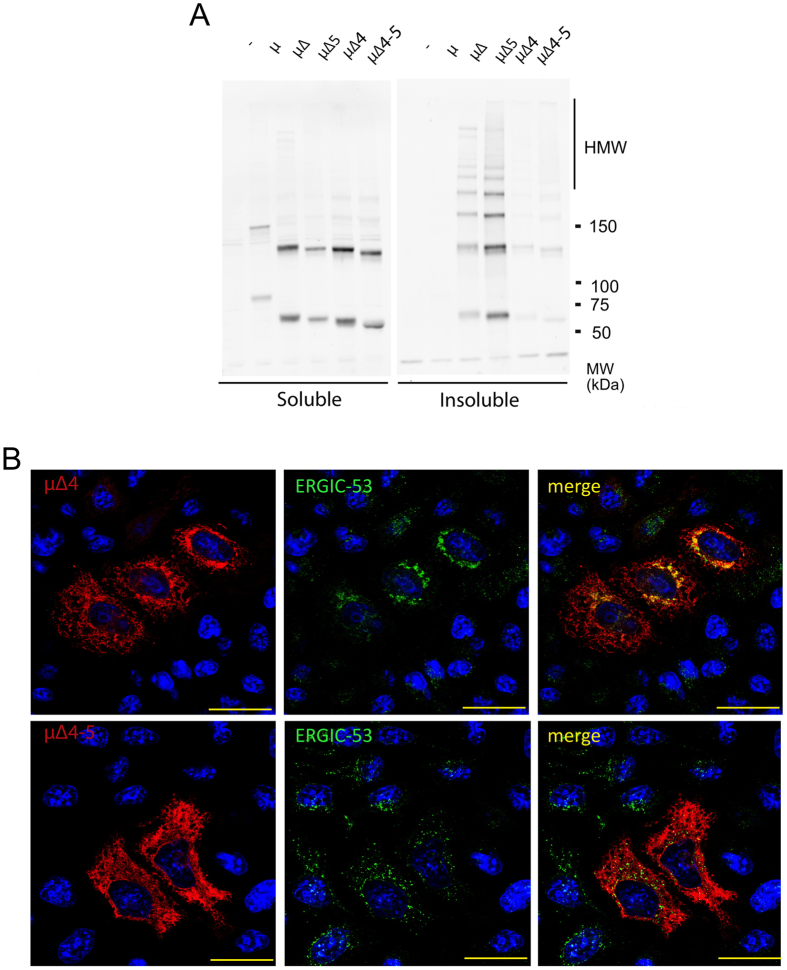
Deleting the N402 glycan prevents μΔ and μΔ5 aggregation. (**A**) HeLa cells were transiently transfected with μ, μΔ, μΔ5, μΔ4 and μΔ5–4 and empty vector as a control (−), as indicated. The NP-40 soluble and insoluble material from 10^5^ cells was resolved under non-reducing conditions and western blots visualized with anti-μ. Clearly, mutating the N402 glycan prevents aggregation, and both μ∆4 and μ∆4–5 accumulate in the soluble fraction mainly as dimers and monomers. (**B**) Forty hours after transfections, HeLa cells transiently transfected with μΔ4 and μΔ4–5 were fixed with PFA and stained with antibodies against μ and ERGIC-53. Note that μΔ4, but not μΔ4–5, co-localizes with ERGIC-53.

The higher avidity and larger size of the aggregates formed by μ∆5 could compensate for the lower affinity of non-processed 402 glycans in recruiting ERGIC-53. To establish whether this was the case, we replaced N402 for glutamine in either μ∆ or μΔ5, to generate the μΔ4 and μΔ4–5 mutants, respectively. Immunofluorescence assays demonstrated that removal of the 402 glycan almost completely prevents aggregation. Both μΔ4 and μΔ4–5 yielded a reticular staining pattern (Fig. 5A) overlapping with ER markers (not shown). Interestingly, ERGIC-53 is still recruited by μΔ4 but not by μΔ4–5 (Fig. 5B), indicating that either sugar can be recognized singularly by the lectin. Biochemical analyses confirmed that, while μΔ and μΔ5 accumulated abundantly in the non-soluble fraction, mutants lacking 402 glycans formed few HMW species and remained soluble (Fig. 5A). Taken together, these findings confirm that *in vivo* the presence of μ4 sugars is important for efficient IgM polymerization^13^ and s(upe)RB formation.

The molecular weight shifts clearly detectable in western blot analyses (Supplementary Figure 2) confirmed that kifunensine inhibited mannose trimming. The mobility differences were attenuated in μΔ5, μΔ4 and above all μΔ4–5 (Supplementary Figure 2). As previously noted in our imaging analyses, kifunensine had little if any effect on the aggregation of μΔ5. Slightly more μΔ4 accumulated in the insoluble fraction upon mannosidase I inhibition: a possible explanation is that under these conditions degradation of this mutant is partly inhibited. However, kifunensine had no effect on the distribution of μ∆4–5 between the soluble and non-soluble fractions.

#### Concluding remarks

**Figure 6:**
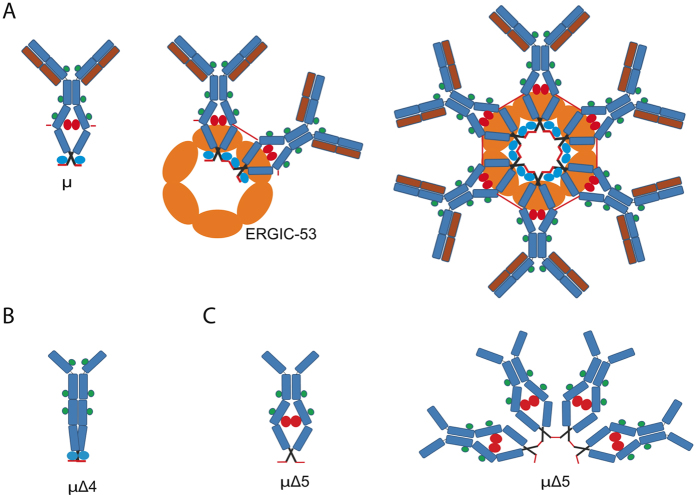
Sweet control of polymerization: the C-terminal μ sugars as spacers and adaptors. (**A**) Interactions with ERGIC-53 favour and control IgM polymerization, the N563-glycan (blue circles) and N402 (red circles) being the main binding sites in μ chains. (**B**) Without the N402 glycan, μ_2_L_2_ subunits may adopt a closed, transport-incompetent conformation, C575 becoming inaccessible for polymerization. (**C**) In the absence of the N563 glycan, high molecular weight polymers are formed, suggesting that this sugar acts as a spacer between adjacent subunits or/and that the interaction with ERGIC-53 only via N402 can cause aberrant polymerization.

So far, no crystallographic data are available for polymeric IgM. Current models predict a mushroom shape with tightly packed Cμ3 and Cμ4 domains. Upon antigen binding, these undergo conformational changes, allowing efficient complement fixation^32^,^33^. Since μ∆ aggregation and polymerization are faces of the same coin, our experiments confirm that the two C-terminal N-glycans (402 and 563) are crucial for IgM biogenesis. They can act in at least two non-alternative ways. Firstly, by engaging ERGIC-53, or additional lectins present in differentiating B cells, so as to time and shape compaction of the mushroom stem. Secondly, they could act as spacers, N402 facilitating the exposure of C414 and C575 and hence polymer formation, N563 limiting instead the number of subunits that can be inserted into planar polymers (Fig. 6). Accordingly, the absence of the 402 glycan is dominant on CH1 deletion, preventing μ∆ aggregation.

Replacing the Cμ1 with different tags (GFP, RFP, Halo) yielded different aggregation and localization patterns (our unpublished results) suggesting that additional factors are in play to assist polymerization *in cis* as well as *in trans*. Considering the biotechnological relevance of a portable polymerization module, further experiments are needed to dissect the intrinsic and extrinsic factors that control IgM biogenesis.

## Materials and Methods

### Cells, plasmids and reagents

Unless otherwise indicated, chemicals were from Sigma Chemical Co (ST. Louis, MO). HeLa, HepG2 and Hek293 cells were obtained from ATCC, Hela-off from Clonthech, and cultured in DMEM (GIBCO Life Technologies) containing 2 mM glutamine and 5% FCS (GIBCO Life Technologies).

Plasmids driving the expression of Ig-λ chain, μΔ and μΔ5 were previously described^11^,^14^,^19^. Plasmids encoding μΔ4 and μΔ4–5 were obtained replacing asparagine 402 for glutamine in μΔ and μΔ5 by site directed mutagenesis with the following primers: Fw-GGAAAGCCATCCCCAAGGCACCTTCAGTG and Rev-CACTGAAGGTGCCTTGGGGATGGCTTTCC. All PCR products were checked by sequencing (GATC Biotech, Milan, Italy).

Rabbit polyclonal anti-calreticulin and anti-ERGIC-53 were purchased from SIGMA Aldrich; goat anti-mouse (IgM) μ-chain antibodies (Alexa 546, 647, 680 and 700) were purchased from Invitrogen; mouse anti-GM130 from BD Transduction Laboratories; the monoclonal AC38 antibody was previously described^14^,^34^. Rabbit ant-p115, rabbit anti-Sec31 and mouse monoclonal anti-ERGIC53 antibodies were kind gifts from Drs. De Matteis (TIGEM, Naples, IT), Hong (Institute of Molecular and Cell Biology, Singapore) Appenzeller-Herzog and Hauri (Biozentrum, University of Basel, Switzerland).

Cells were transfected using calcium phosphate or polyethylene imine (PEI, Polysciences, Inc.) or silenced with specific siRNAs targeted by Lipofectamine RNAimax (Invitrogen, Eugene, Oregon, USA), following the manufacturers’ instructions. ERp44 duplexes sequences were previously described^19^.

### Immunofluorescence

Cells were cultured and transfected on glass coverslips. 48 hours after transfection, cells were fixed with 4% paraformaldehyde for 10 min at RT, permeabilized with PBS containing 0.1% Tx100 for 5 min at RT, washed in PBS, saturated with 2% FCS and then stained with the indicated primary and secondary antibody as described^19^. Slides were mounted in 90% glycerol and images acquired with an Olympus inverted fluorescence microscope (model IX70) with DeltaVision RT Deconvolution System (Alembic, HSR). Deconvoluted images were processed with Adobe Photoshop 7.0 (Adobe Systems Inc.). In other cases, images were taken with a Leica TCS SP2 laser Scanning Confocal Microscope.

### Electron microscopy

For cryo-electron microscopy, HeLa cells expressing μΔ or μΔ5 were fixed for 1 hour at room temperature (0.2% glutaraldehyde/2% paraformaldehyde in cacodylate Buffer 0.1 M) and processed as described^35^. Briefly, samples were embedded in 12% gelatin, infiltrated in 2.3 M sucrose and frozen in liquid nitrogen. Cryosections were obtained using a Leica EM FC7 ultramicrotome (Leica microsystem, Vienna, Austria) and collected on 150 mesh formvar carbon coated copper grids. Grids were then incubated with 0.1 μg/μl rabbit anti-μ (Zymed Laboratories, San Francisco, CA) followed by goat anti-rabbit IgG coupled to 15 nm gold beads.

Grids were contrasted in a solution of uranyl acetate and methylcellulose, air-dried and observed in a Leo 912AB transmission electron microscope (Carl Zeiss, Oberkochen, Germany).

Images were analysed with ImageJ in order to determine the size of the μ-containing vesicles. At least two perpendicular measurements were performed for each structure; 60 structures were analysed for each sample and the diameter averaged.

### Cell Lysis and Western Blotting

48 hours after transfection, cells were washed and lysed at the concentration of 1 × 10^4^ cells/μl in buffer A (0.2% Tx100, 50 mM Tris-HCl pH 7.5), 150 mM NaCl, 5 mM EDTA, 10 mM *N-*ethylmaleimide and a cocktail of protease inhibitors (Roche, San Francisco, CA, USA). The Tx100-insoluble fraction (insol) was separated by centrifugation at 3,400 g for 10 minutes and solubilized in lysis buffer B (1% SDS, 50 mM Tris-HCl pH 7.5, 10 mM NEM) for 10 minutes at RT, diluted in 50 mM Tris-HCl pH 7.5, 0.2% Tx100, to keep the volume of the soluble and insoluble fractions equal, and sonicated for 10 seconds. In order to collect the secreted material, 48 hours after transfection, cells were washed three times with PBS and incubated for 4 hours in pre-warmed OPTIMEM. After 4 hours, cell culture supernatant was collected (SN), cells were detached from the plate with PBS containing 10 mM EDTA. The secreted material attached to the plate (plate) was then scraped from the plate in 2% SDS, 0.1 M Tris pH 7.4, 10 mM *N-*ethylmaleimide and a cocktail of protease inhibitors (Roche, San Francisco, CA, USA). Samples were resolved under reducing or non-reducing conditions by pre-casted 10% or 4–12% acrylamide gradient gels (Invitrogen, Eugene, Oregon, USA). After transfer to nitrocellulose and decoration with specific antibodies, images were acquired with the fluorescence Scanner Fuji FLA 9000 (FujiFilm Life Science, Tokyo, Japan) and processed with Adobe Photoshop 7.0 (Adobe Systems Inc.). For densitometric quantification, WB images were analyzed with Image J.

## Additional Information

**How to cite this article**: Giannone, C. *et al*. Roles of N-glycans in the polymerization-dependent aggregation of mutant Ig-µ chains in the early secretory pathway. *Sci. Rep.* **7**, 41815; doi: 10.1038/srep41815 (2017).

**Publisher's note:** Springer Nature remains neutral with regard to jurisdictional claims in published maps and institutional affiliations.

## Supplementary Material

Supplementary Information

## Figures and Tables

**Figure 1:**
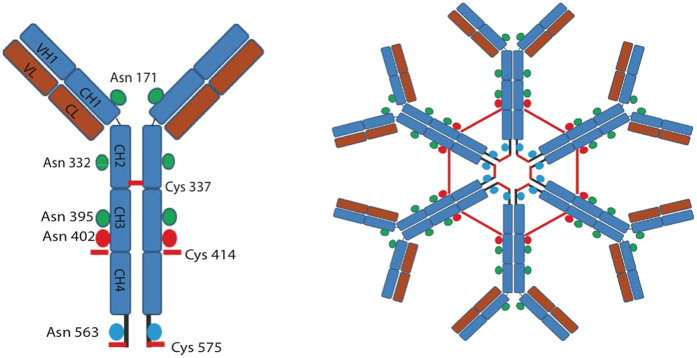
Schematic representation of IgM monomers and hexamers. Ig-μ and Ig-L chains are schematized in blue and red, respectively. The five N-glycans of Ig-μ are indicated as coloured spheres: in green the three glycans that undergo Golgi processing. In red and blue the two N-glycans that are Endo-H sensitive also in secreted IgM polymers (402 and 563, respectively). The two cysteines involved in the formation of the disulphide bonds between adjacent IgM monomers (414 and 575) are indicated as short red lines. Cysteine 337 forms an inter-chain disulfide linking two Cμ2 within μ_2_L_2_ ‘monomers’. On the right the arrangement of planar IgM hexamers formed in the absence of Ig-J chains^5^ is shown.
