# Supplementary material for: Roles of N-glycans in the polymerization-dependent aggregation of mutant Ig-μ chains in the early secretory pathway
Source: Sci Rep. 2017 Feb 3;7:41815. doi: 10.1038/srep41815 (PMC5291101; doi:10.1038/srep41815)
Supplement: Supplementary Information [file srep41815-s1.pdf]

SREP-16-29083A Giannone, Fagioli, Valetti, Sitia & Anelli. Roles of N-glycans in the polymerization-dependent aggregation of mutant Ig-μ chains in the early secretory pathway

Supplementary Figure 1 μΔ and μΔ4 do not co-localize with p115

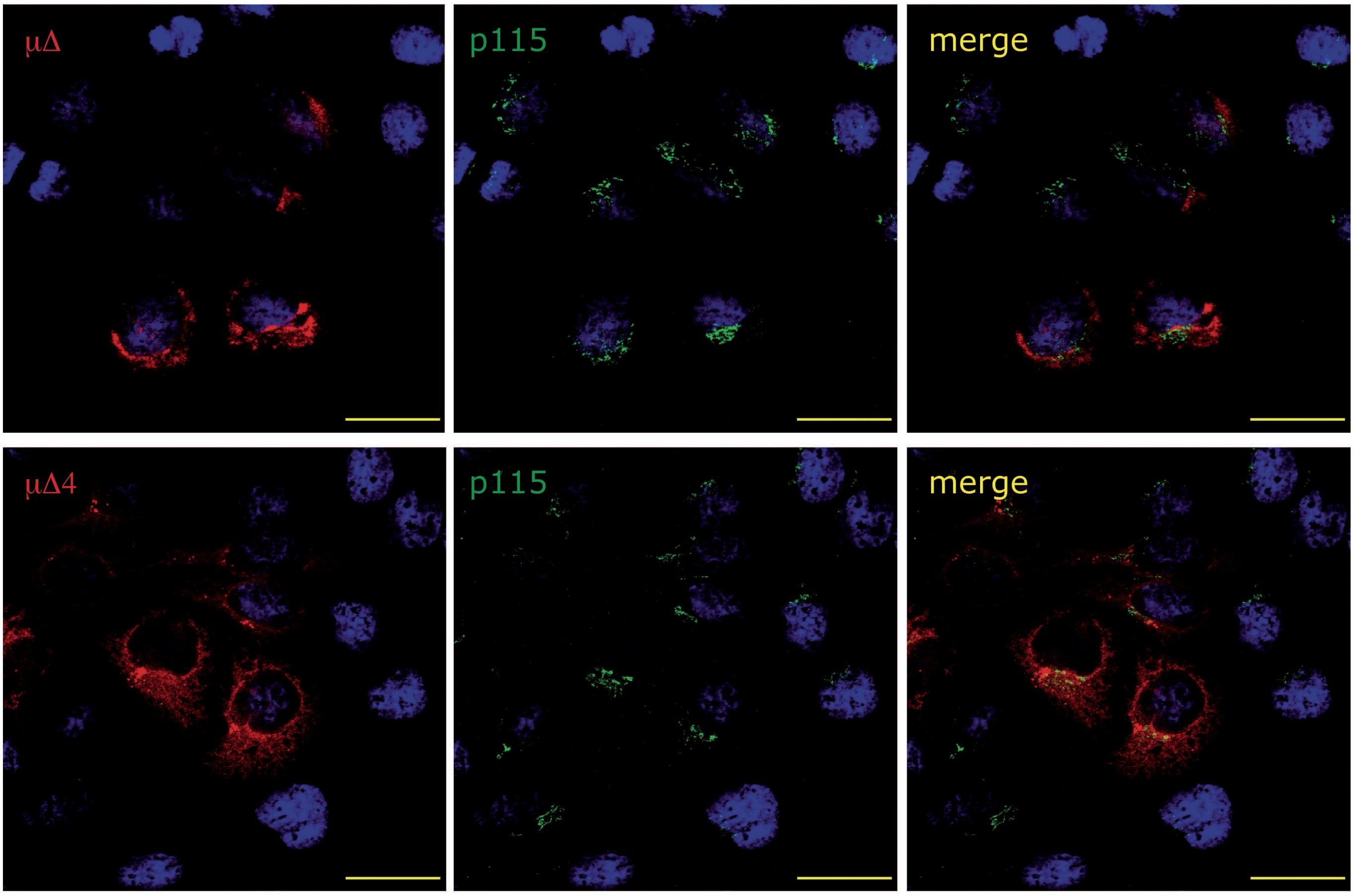

HeLa cells transiently transfected with μΔ or μΔ4 were fixed with PFA and stained with antibodies against p115 (green) and Ig-μ (red): clearly, no co-localization is visible (bar: 15μm)

Supplementary Figure 2. μΔ4 and μΔ4-5 display a reticular pattern and are largely detergent- soluble

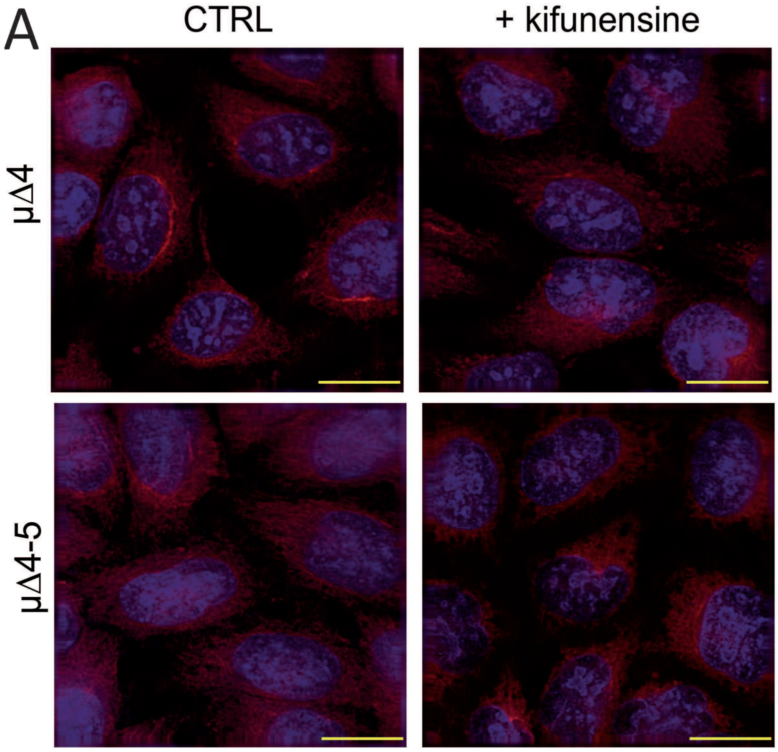

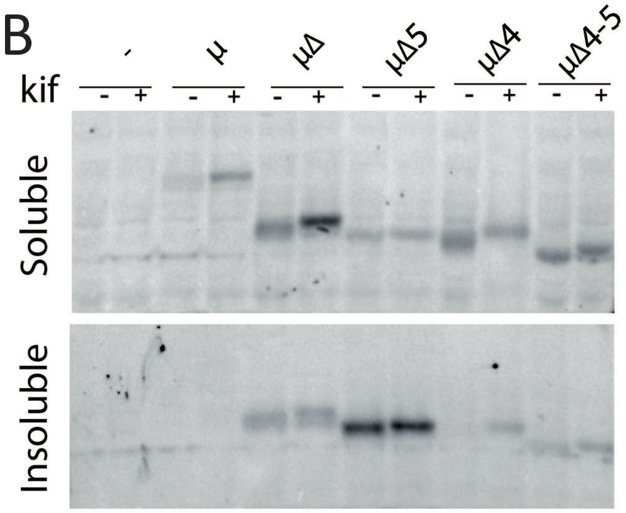

A HeLa cells transiently transfected with μΔ4 or the double mutant μΔ4-5 were treated with or without kifunensine and processed as in Figure 4B. Both transfectants display a reticular pattern that is not modified by kifunensine (bar: 15 μm).

B Aliquots of the detergent-soluble and insoluble extracts from μΔ, μ4, μΔ5 and μΔ 5-4 expressing cells treated with or without kifunensine were resolved under reducing conditions. Preventing ER mannosidase I activity increased the solubility of μΔ but only minor effects were observed on μΔ5, μΔ4 and μΔ4-5.
